# The rostro-caudal gradient in the prefrontal cortex and its modulation by subthalamic deep brain stimulation in Parkinson’s disease

**DOI:** 10.1038/s41598-021-81535-7

**Published:** 2021-01-22

**Authors:** F. Konrad Schumacher, Lena V. Schumacher, Florian Amtage, Andreas Horn, Karl Egger, Tobias Piroth, Cornelius Weiller, Björn O. Schelter, Volker A. Coenen, Christoph P. Kaller

**Affiliations:** 1 Department of Neurology, Medical Center, University of Freiburg, Breisacher Str. 64, 79106 Freiburg, Germany; 2 Department of Neuroradiology, Medical Center, University of Freiburg, Freiburg, Germany; 3 Freiburg Brain Imaging Center, University of Freiburg, Freiburg, Germany; 4 Faculty of Biology, University of Freiburg, Freiburg, Germany; 5 Medical Psychology and Medical Sociology, University of Freiburg, Freiburg, Germany; 6 Department of Neurology, Movement Disorders and Neuromodulation Unit, Charité, University Medicine Berlin, Berlin, Germany; 7 Kantonsspital Aarau, Aarau, Switzerland; 8 Institute for Complex Systems and Mathematical Biology, University of Aberdeen, Aberdeen, UK; 9 Department of Stereotactic and Functional Neurosurgery, Medical Center, University of Freiburg, Freiburg, Germany; 10 Faculty of Medicine, University of Freiburg, Freiburg, Germany; 11 BrainLinks-BrainTools Cluster of Excellence, University of Freiburg, Freiburg, Germany

**Keywords:** Parkinson's disease, Cognitive control

## Abstract

Deep brain stimulation of the subthalamic nucleus (STN-DBS) alleviates motor symptoms in Parkinson’s disease (PD) but also affects the prefrontal cortex (PFC), potentially leading to cognitive side effects. The present study tested alterations within the rostro-caudal hierarchy of neural processing in the PFC induced by STN-DBS in PD. Granger-causality analyses of fast functional near-infrared spectroscopy (fNIRS) measurements were used to infer directed functional connectivity from intrinsic PFC activity in 24 PD patients treated with STN-DBS. Functional connectivity was assessed ON stimulation, in steady-state OFF stimulation and immediately after the stimulator was switched ON again. Results revealed that STN-DBS significantly enhanced the rostro-caudal hierarchical organization of the PFC in patients who had undergone implantation early in the course of the disease, whereas it attenuated the rostro-caudal hierarchy in late-implanted patients. Most crucially, this systematic network effect of STN-DBS was reproducible in the second ON stimulation measurement. Supplemental analyses demonstrated the significance of prefrontal networks for cognitive functions in patients and matched healthy controls. These findings show that the modulation of prefrontal functional networks by STN-DBS is dependent on the disease duration before DBS implantation and suggest a neurophysiological mechanism underlying the side effects on prefrontally-guided cognitive functions observed under STN-DBS.

## Introduction

Many aspects of human behavior rely on the prefrontal cortex (PFC) and its interactions with a variety of other cortical and subcortical brain structures^1^. The PFC is directly connected to the subthalamic nucleus (STN)^2–4^, a prime target of deep brain stimulation (DBS) in Parkinson’s disease. Although STN-DBS is primarily used to target the motor circuit of cortex-basal ganglia interactions, changes of activity and behavior in associative loops have been widely observed, studied and described^5–8^. Specifically, STN-DBS intervenes in a complex network of cortico-subcortical pathways, providing a variety of potential routes to interfere with remote brain areas^9^. Given that STN-DBS is assumed to shift the balance between inhibitory and excitatory network activity to restore functionality of the diseased motor system^10,11^, currents spreading beyond the stimulation target can likely compromise the balance in associative and limbic cortico-basal ganglia loops connected with the STN^12^. Although the benefits of STN-DBS for the alleviation of motor symptoms are unquestioned^13–15^, cognitive and psychiatric side effects like impaired verbal fluency^16–18^, impulsive behavior^19^ and even a possibly increased suicidal tendency^20^ have been reported. These impairments indicate far-reaching implications of modulating basal-ganglia networks, suggesting that STN-DBS may impact on the functional integrity of the PFC^12,21^. Directly assessing DBS-induced neurophysiological changes in the PFC may hence substantially advance our understanding of remote effects beyond the targeted stimulation sites.

Several models of human PFC functioning have suggested a rostro-caudal hierarchical organization^22–24^. In these, it is assumed that information processing in rostral PFC precedes and determines processing in caudal PFC^23^. Indeed, as shown by functional magnetic resonance imaging (fMRI) and lesion studies, rostral parts of the lateral PFC get gradually more involved when cognitive demands become increasingly abstract, interdependent, and temporally extended^25–27^. Moreover, recent evidence suggests that the upper end of this processing cascade resides in the mid-dorsolateral PFC rather than in the frontal pole^28–31^.

The PFC directly projects into the STN by the associative hyperdirect pathway: Fibers of the corticospinal tract send axon collaterals to the associative part of the nucleus^3,32,33^. Moreover, with disease progression, frontal areas involved in both motor and associative loops show increased atrophy which has recently been linked to the degree of clinical improvement of Parkinson’s patients under STN-DBS^34^. Thus, the outcome of STN-DBS significantly relies on the integrity of the PFC, which is possibly influenced by the stimulation of the limbic hyperdirect pathway that was recently found to be confluent with the superolateral medial forebrain bundle in humans^2^. In consequence, the remote impact of STN-DBS on cortical regions is likely to differ between Parkinson’s patients that undergo surgery early and late after disease onset.

Based on the assumption of rostro-caudally directed interactions within the PFC^22–24^, we therefore directly assessed STN-DBS effects on the integrity of this prefrontal hierarchical network by comparing the rostro-caudal gradient of neural processing in the lateral PFC, estimated from intra-individual functional measurements of Parkinson’s patients in different DBS states—initially in the ON state, followed by steady-state OFF, and thereafter again ON DBS. To this end, we used a recently established approach on estimating directed functional connectivity based on multi-channel near-infrared spectroscopy (fNIRS) measurements that provide a high temporal and a sufficient spatial resolution to reveal the spatiotemporal evolution of directed neural activity within the lateral PFC (e.g. Refs^31,35,36^). Thus, the present approach evaluates the functional integrity of those cortical regions that are fundamental for higher-order cognitive functions. In addition, neuropsychological assessments were used to establish a link between the estimated prefrontal hierarchical network properties and cognitive functioning.

## Results

**Figure 1:**
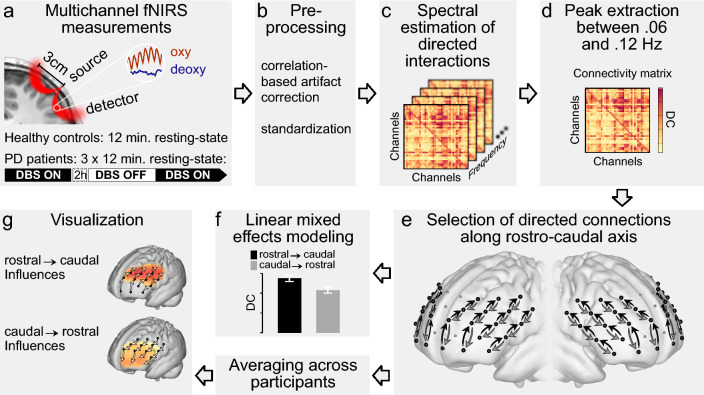
Scheme of the analysis workflow followed in the present study. Patients watched muted parts of a nature documentary during fNIRS measurements (**a**). Artifact correction^67^ was applied, resulting in perfectly anticorrelated signals of oxygenated and deoxygenated hemoglobin (**b**). The time-series were standardized before estimation of directed coherence (DC) as a measure of directed interactions between fNIRS channels^69^ (**c**). The maximum DC value in the frequency band between 0.06 and 0.12 Hz was extracted to yield a 2-dimensional, directed connectivity matrix for each measurement (**d**). According to models postulating a predominant flow of information from rostral to caudal PFC the influences between PFC regions should be significantly stronger in rostral-to-caudal direction than in caudal-to-rostral direction. This prediction was tested by analyzing connections between directly neighboring channels along four rostro-caudal streams comprising four channels each (black and gray arrows in panel e) and covering most of the lateral surface of the PFC in each hemisphere (**e**). The distinction between rostro-caudal and caudo-rostral connections was represented in the model by the factor *direction of influences* (**f**). Influences in both directions were averaged across patients and projected separately on a standard brain surface (**g**).

The analysis workflow, including the measurement protocol and illustration of fNIRS channel positions, is summarized in Fig. 1. The main analyses were conducted by means of two linear mixed-effects models; the first model investigated whether STN-DBS (compared ON vs. OFF) exerted an influence on the rostro-caudal connectivity in the lateral PFC, while the second model (additionally including data from the second ON measurement, cf. Figure 1A) served as an experimental validation of the main finding of a stimulation-induced alteration of the rostro-caudal connectivity. An overview of the fixed and random effects structures of Models 1 and 2 is summarized in Table 1. The concise report of the results below is complemented by a comprehensive report of the respective statistical indices underlying significant effects, estimates, and contrasts in the Supplementary Table S2. Additionally, control analyses including healthy adults (Supplementary Model S1, Supplementary Fig. S2), assessing the role of the rostro-caudal gradient for cognitive functions (Supplementary Model S2, Supplementary Fig. S3), and investigating the role of gray matter volume as a proxy of disease-induced cortical atrophy for directed functional connectivity (Supplementary Model S3) are available in the Supplementary Information.

**Table 1:** Initial fixed and random effects structure of each model.

Model	Fixed effects terms	Random intercepts
1: ON and OFF measurements	dir. × hemisphere × stim. × age at disease onset + dir. × hemisphere × stim. × disease duration before DBS implantation + dir. × hemisphere × stim. × time since DBS implantation + dir. × hemisphere × stim. × LEDD + dir. × hemisphere × stim. × VAT	Stream × participant Level × participant
2: ON, OFF & 2nd ON measurements	dir. × stim. × disease duration before DBS implantation	Stream × participant Level × participant

N.B. Exhaustive models were successively reduced by non-significant higher-order terms to yield a parsimonious model (see Supplementary Table S2 for significant effects). Continuous predictors are underlined. All lower-order terms were included. Random effects were not reduced.

*dir.* direction of influences, *LEDD* levodopa equivalent daily dose, *stim.* stimulation state (ON, OFF, and, in model 2, 2nd ON), *VAT* volume of activated tissue.

### STN-DBS modulates hierarchical processing in the PFC (Model 1)

In Model 1 we tested the hypothesis that STN-DBS has an impact on rostro-caudally directed interactions in the PFC. Understanding remote effects of STN-DBS is however challenged by the multitude of variables that add to the outcome of the treatment, including e.g. the patient’s preoperative clinical status^14^, stimulation parameters^37^, electrode positions^38^, and concomitant drug treatment^39,40^. Moreover, higher age and longer disease duration have been discussed to be disadvantageous for the clinical outcome of STN-DBS^34^. The initial fixed effects structure of model 1 (Table 1) therefore included the following factors of interest: *stimulation state* (ON vs. OFF), *direction of influences* (rostro-caudal vs. caudo-rostral), and *hemisphere* (ipsilateral vs. contralateral with respect to the hemisphere of disease onset) and all possible interactions between this set of within-subject-factors as well as each of the following covariates: (I) age at disease onset (initial diagnosis), (II) disease duration before DBS implantation, (III) time since DBS implantation (I–III summing up to the chronological age), (IV) dopaminergic medication in terms of the levodopa equivalent daily dose (LEDD, calculated according to Tomlinson et al.^41^), and (V) stimulation intensity in terms of the volume of activated tissue (VAT). This extensive model was successively reduced by non-significant fixed effects terms to yield a parsimonious model^42^. Multicollinearity was controlled using the variance inflation factor (VIF, all VIF < 3.6)^43,44^. Correlation coefficients for the covariates are provided in Supplementary Table S3.

**Figure 2:**
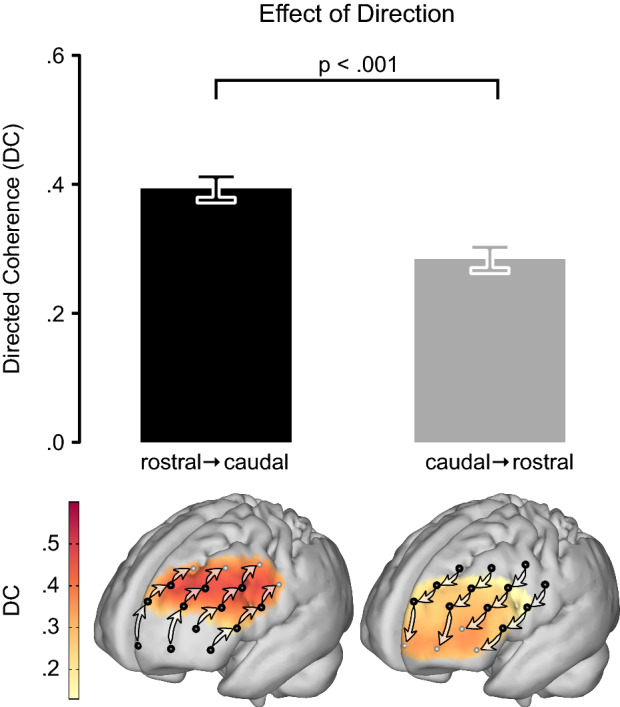
Granger-causality analysis of directed interactions reveals the rostro-caudal hierarchical organization in the PFC. The significant main effect for *direction* in the mixed effects model showed that influences from rostral to caudal PFC were stronger than from caudal to rostral (top panel). For cortical projections (bottom panel), directed coherence (DC) values were averaged across hemispheres and projected onto the cortical surface to represent the influences from channels (black dots) toward caudally (left brain) and rostrally (right brain) neighboring channels as indicated by arrows. Darker red colors signify stronger influences in terms of higher DC values. Bars in the top panel represent least square means; error bars indicate 95% confidence intervals; n = 24.

**Figure 3:**
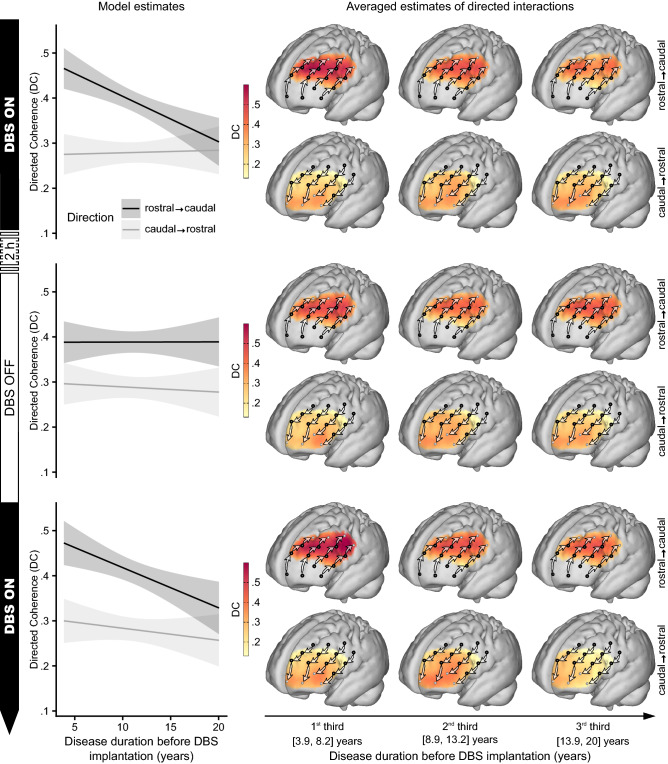
The effect of DBS on rostro-caudally directed interactions in the PFC depends on the disease duration before DBS implantation. The relationship between the rostro-caudal gradient and the *disease duration before DBS implantation* in the ON stimulation state (top row, n = 24) shows that patients who received DBS early in the course of their disease had a strong gradient that was even stronger than in the OFF stimulation state (middle row, n = 22). The later in the individual course of the disease patients underwent implantation, the more influences in rostro-caudal direction declined, such that patients who received DBS late after disease onset had a clearly diminished gradient ON stimulation compared to OFF stimulation. The replication of this time-dependent impact of DBS stimulation on the rostro-caudal hierarchical organization of the PFC in the second ON stimulation measurement (bottom row, n = 18) further confirmed this dependence (Model 2, see below). The projection of directed coherence (DC) values on the cortical surface was done analogous to Fig. 2 but separately for three sub-groups, split at the terciles of the *disease duration before DBS implantation*. Model predictions are plotted with 95% non-simultaneous confidence bands.

Confirming the assumed rostro-caudal hierarchical organization of the PFC, the significant main effect for direction revealed that influences in rostro-caudal direction were generally higher than caudo-rostral influences (F(1,1995.4) = 110.9, p < 0.001, Fig. 2). As STN-DBS is known to have side effects on cognitive functions subserved by the PFC^16,19^, we hypothesized that the prefrontal rostro-caudal gradient could be compromised ON compared to OFF stimulation. While the respective two-way interaction between *stimulation state* (ON vs. OFF) and the *direction of influences* (rostro-caudal/caudo-rostral) was not statistically significant on its own (F(1,1995.4) = 0.6, p = 0.454), it was dependent on the *disease duration before DBS implantation* (F(1,1995.4) = 5.8, p = 0.016). This three-way interaction was primarily driven by influences in rostro-caudal direction (Fig. 3 top row, Supplementary Table S2): Patients who underwent DBS implantation early after disease onset showed stronger rostro-caudal influences and a steeper gradient ON compared to OFF stimulation. In contrast, in patients who received DBS implantation at longer disease durations the rostro-caudal gradient was diminished ON stimulation. Moreover, there was no significant relation between the *disease duration before DBS implantation* and the gradient in the OFF state (Fig. 3 middle row).

**Figure 4:**
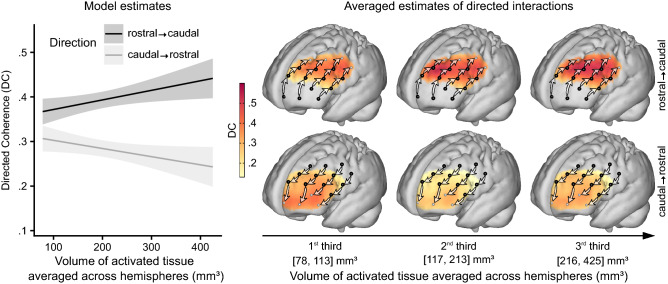
Rostro-caudally directed interactions in the PFC increase with stimulation intensity. The prediction by the linear mixed effects model showed a steep increase of influences from rostral to caudal prefrontal cortex (PFC) (black line) with larger *volumes of activated tissue* (*VAT*) and stimulation strength. At the same time, caudo-rostrally directed influences weakened with stimulation strength (gray line). These diverging trends can also be observed from the projected directed coherence (DC) values, where colors indicate the strength of influences between neighboring channels in rostro-caudal (top row) and caudo-rostral direction (bottom row). Sources of influences are marked by black dots. The projection of DC values was done separately for three sub-groups, split at the terciles of *VAT*. The most striking difference between patients with small and large VATs appears in influences exerted by the mid-lateral on the caudal PFC. Model predictions in the left panel are shown with 95% non-simultaneous confidence bands; n = 23.

Model 1 further revealed that the prefrontal gradient heavily depended on the stimulation intensity (F(1,1995.4) = 15.6, p < 0.001), with a larger *VAT* being associated with both stronger rostro-caudal and weaker caudo-rostral influences (Fig. 4, Supplementary Table S2). This two-way interaction between *VAT* and the *direction of influences* was independent of whether STN-DBS was turned ON or OFF and persisted after stimulation was switched off for 2 h.

**Figure 5:**
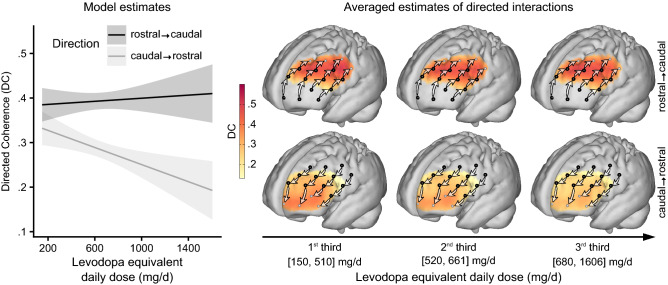
Dopaminergic medication increases the rostro-caudal gradient in the PFC by diminishing rostrally directed influences. The enhancing effect of the *levodopa equivalent daily dose* (*LEDD*) on the rostro-caudal gradient was mainly driven by a strong decrease of influences in caudo-rostral direction (bottom row of projections). Analogous to Figs. 3 and 4, the projection of directed coherence (DC) values was done separately for three sub-groups, split at the terciles of *LEDD*. Model predictions are plotted with 95% non-simultaneous confidence bands; n = 24.

Besides *VAT*, *LEDD* was also positively associated with the strength of the rostro-caudal gradient (F(1,1995.4) = 9.6, p = 0.002). However, this two-way interaction between *LEDD* and the *direction of influences* was mostly driven by the simple effect of influences in one direction, i.e. *LEDD* was negatively correlated with caudo-rostrally directed influences, while the relationship between influences in rostro-caudal direction and *LEDD* failed to reach significance (Fig. 5, Supplementary Table S2).

Albeit weak, a third two-way interaction was present between *hemisphere* and the *direction of influences* (F(1,1995.4) = 4.6, p = 0.033). The rostro-caudal gradient appeared to be steeper in the ipsilateral than in the contralateral hemisphere with respect to the hemisphere of disease onset. This within-patient effect hence corroborates the enhancing between-patient effect of the *VAT* on the rostro-caudal gradient, as *VAT*s were larger in the ipsilateral than in the contralateral hemisphere (mean *VAT* in ipsilateral hemisphere: 230 mm^3^; mean *VAT* in contralateral hemisphere: 174 mm^3^, t(22) = 2.0, p = 0.053). Thus, the hemispheric differences in the gradient may be introduced by the asymmetric stimulation intensities.

Taken together, the key finding of Model 1 constituted a significant modulation effect of STN-DBS on the rostro-caudally directed interactions in the PFC that was moderated by the patients’ *disease duration before DBS implantation.* Given that *disease duration before DBS implantation* was highly correlated with the *overall disease duration* (Pearson’s r = 0.79, p < 0.001), we conducted a control analysis and refitted the model with (I) the variables *disease duration before DBS implantation* and *time since DBS implantation* substituted by the *overall disease duration* and (II) with all three time-related covariates (*age at disease onset*, *disease duration before DBS implantation* and *time since DBS implantation*) substituted by *chronological age*. Neither *overall disease duration* nor *chronological age* exerted any significant moderator effect (all p > 0.05) on the directed interactions in the PFC, demonstrating that the impact of STN-DBS on the rostro-caudal gradient was indeed specifically dependent on the *disease duration before DBS implantation*.

### Remote effects of STN-DBS on PFC are reproducible (Model 2)

As an experimental validation, in Model 2 we addressed the reproducibility of the modulation effect of STN-DBS on the rostro-caudally directed interactions in the PFC and its dependence on the *disease duration before DBS implantation*. To this end, the examination followed a fixed protocol with the initial ON measurements always being followed by the subsequent OFF measurements. Precluding that the stimulation-dependent results in Model 1 were simply driven by carry-over effects would hence require to demonstrate that the dependence of the STN-DBS effects on the *disease duration before DBS implantation* was reversible and would reappear after stimulation was switched on again. In consequence, we fitted Model 2 to the data of the ON, OFF, and ON2 measurements and, as a fixed effect, included the respective three-way interaction between *stimulation state*, *direction of influences* and *disease duration before DBS implantation* as well as all corresponding lower-order terms. As depicted in Fig. 3, the negative correlation between the *disease duration before DBS implantation* and the rostro-caudal gradient was present in the ON state and absent in the OFF state. Most importantly, it reemerged in the ON2 state (Fig. 3, bottom row), within only a few minutes after the stimulation was switched on again. As the three-way interaction closely resembled the pattern observed in Model 1, it hence demonstrated its immediate reproducibility (F(2,2949.6) = 3.2, p = 0.041). Post-hoc comparisons further confirmed that for influences in rostro-caudal direction the effect of *disease duration before DBS implantation* in the ON and ON2 state was significantly different from the OFF state, while there was no significant difference between the ON and the ON2 state (Fig. 3, Supplementary Table S2). Regarding caudo-rostral influences, there was no significant simple effect of *disease duration before DBS implantation* in any *stimulation state*.

In summary, the modulation of directed influences between PFC sub-regions by STN-DBS and its moderation by *disease duration before DBS implantation* was not only replicable, but also specific to the predominant rostro-caudal direction within the PFC and specific for ON states, arguing for a significant stimulation-induced network effect of STN-DBS on PFC integrity.

### Supplemental control analyses

Healthy participants that were matched to PD patients in terms of age and sex (Supplementary Fig. S1) were assessed in two supplemental analyses. Supplementary Model S1 revealed that the rostro-caudal connectivity gradient was also apparent in healthy controls (Supplementary Fig. S2). Supplementary Model S2 included both PD patients and matched healthy controls and demonstrated that the resting-state connectivity gradient within the rostral PFC was associated with performance in a prefrontally-guided cognitive planning task as well as a more general measure of global cognitive ability (Supplementary Fig. S3). Thus, the present approach consistently detects a rostro-caudal functional network in the PFC that underlies higher cognitive abilities. Another supplemental control analysis testing the predictability of the directed functional connectivity by gray matter volume in the PFC revealed no significant interaction effects, precluding that the variance in the rostro-caudal gradients within the functional networks was significantly driven by differences in cortical atrophy (Supplementary Model S3).

## Discussion

The present study revealed that STN-DBS in Parkinson’s patients significantly modulates the hierarchical organization of the PFC. Most crucially, this remote network effects of STN-DBS on the PFC depended on disease progression and hence possibly on the level of sustained integrity of nigro-striatal and fronto-striatal circuits: Patients who underwent DBS implantation earlier after disease onset had a stronger rostro-caudal gradient of directed interactions ON versus OFF stimulation. In contrast, patients who received a DBS implantation after longer disease durations showed diminished rostro-caudally directed interactions ON compared to OFF stimulation. Importantly, this effect was clearly reproducible: In the transition when stimulation was switched ON again after steady-state OFF, the dependence of the rostro-caudal gradient in the PFC on the disease duration before DBS implantation re-emerged in the same systematic fashion as before (Fig. 3).

### The role of the basal ganglia for the rostro-caudal hierarchy in the PFC

One hypothesis is that the STN—as a major input nucleus of the basal ganglia—plays an important role in integrating sensorimotor information and regulating the activity of two opposing signaling pathways: the direct, movement-facilitating pathway and the indirect, movement-inhibiting pathway^9,45^. Loss of dopaminergic neurons in the substantia nigra pars compacta in Parkinson’s disease leads to an excessive activity of the STN and an imbalance in favor of the indirect pathway causing hypokinetic symptoms^9^. DBS is known to elicit action potentials in afferent and efferent axons^37^ thus not only exciting downstream neurons in basal ganglia output nuclei but also anti-dromically modulating cortical neurons projecting (as part of the hyperdirect pathway) to the STN^46^. Although the STN was previously assumed to receive cortical projections only from motor cortices^47^ a recent tracer study in macaques revealed topographic projections from various PFC regions (including the dorsolateral PFC) to the dorsomedially located associative functional zone of the STN^48^ which could also be reproduced in human imaging studies^2,3,49^. Antidromic activation of the limbic hyperdirect pathway may therefore contribute to the present stimulation-induced changes in the hierarchical organization of the PFC, affecting cognitive functions^4,50^. Alternatively, remote STN-DBS effects on the PFC may be mediated through the downstream nigro-thalamocortical pathway^37^—in particular as the impact of STN-DBS on rostro-caudally directed prefrontal interactions depended on the disease duration before DBS implantation and therefore on the disease progression at the time STN-DBS was initially administered. In Parkinson’s disease dopamine depletion often progresses from sensorimotor towards associative/cognitive basal ganglia-thalamocortical circuits and affects cortical afferents in caudal PFC earlier than in rostral PFC^5,39^. The enhancement of directed interactions within lateral PFC by STN-DBS may thus be mediated through the intact associative circuits in early-stage Parkinson’s disease, while STN-DBS has debilitating effects on PFC integrity as soon as disease progression has reached associative circuits.

### Disease progression impacts on prefrontal effects of STN-DBS

The present results provide new, complementary evidence suggesting that implantation at an earlier stage of Parkinson’s disease may reduce the risk of DBS-induced detrimental cognitive and psychiatric side effects on prefrontal functioning. As the STN-DBS effect on directed prefrontal interactions depended neither on the duration of stimulation, nor on the overall disease duration, nor on the chronological age but only on the disease duration before DBS implantation, this raises the question whether STN-DBS itself or the reduction of dopaminergic medication following STN-DBS may exert preserving effects on the associative basal ganglia-thalamocortical loops^51^. The stimulation-induced decrease of the rostro-caudal gradient with increasing disease duration before DBS implantation suggests that STN-DBS intervention at early-stage Parkinson’s disease may contribute to sustained PFC integrity and that the timing of implantation is of essence for the overall outcome of DBS^52^. When patients notice first symptoms of Parkinson’s disease, at least 50% of neurons in the substantia nigra are already lost^53^. Animal models of Parkinson’s disease further show that STN-DBS can have neuroprotective effects in the substantia nigra^51^. Although not confirmed in humans so far^52^, it has been discussed that DBS implantation may have a slowing effect on the progression of neurodegeneration^51^. Finally, a brain in a healthier state may simply cope with and adapt to an extensive intervention like DBS better than a more depleted one as, for instance, the motor, cognitive, and psychiatric symptoms of Parkinson’s disease are alleviated by STN-DBS when applied early in the course of the disease^13,15,52^. In line with this, younger patients are known to recover better and have less cognitive decline after surgery than the elderly^54^.

As STN-DBS electrodes were implanted through prefrontal entry points the penetration of the PFC during STN-DBS surgery may also impact on PFC network integrity. However, the present moderation of the rostro-caudal gradient by disease duration before DBS implantation was reversible and only present under active stimulation. It is hence unlikely to be introduced by the surgery itself. Nevertheless, the lesion of the STN caused by electrode implantation is known to have a temporary effect that clinically mimics the effect of stimulation^55^. This so-called stun effect is a transient phenomenon which is apparent within the first postoperative weeks but usually regresses before initial activation of the stimulator. Preliminary results from a follow-up study indicate that the stun effect caused by STN-DBS surgery indeed attenuates the rostro-caudal gradient post- compared to pre-DBS surgery only in patients with long disease durations but not in patients with short disease durations (F. K. Schumacher, V. A. Coenen, C. P. Kaller, unpublished data) reproducing the moderating effect of disease duration before DBS implantation on the rostro-caudal gradient ON stimulation in the current study.

### Effects of stimulation intensities and dopamine level

VAT and LEDD both had a general amplifying effect on the strength of the prefrontal gradient. However, the effect of VAT was driven by both increasing rostro-caudal influences and decreasing caudo-rostral influences, while the effect of LEDD solely relied on decreasing influences in caudo-rostral direction (Figs. 4, 5). The mechanisms behind both effects are hence likely to be distinct^40^. Moreover, shifts in network states induced by the systemic application of dopamine are mainly dosage dependent, whereas the VAT effect on cortical networks is probably transduced through parts of the basal ganglia-cortical loops and may also rely on resonance mediated by the direct, indirect, and limbic hyperdirect pathway^2,45^.

Interpreting the effects of VAT and LEDD is limited by the lack of data from patients OFF medication or before DBS implantation. A stronger gradient may in fact be the cause for rather than the result of stronger stimulation amplitudes (i.e. patients having a stronger gradient might need a stronger stimulation to alleviate motor symptoms). The persistent VAT effect OFF stimulation may support this argument but it may likewise reflect plastic changes in network organization or sustained shifts in neurochemical homeostasis^56^.

Finally, the magnitude of the VAT estimates (Supplementary Table S1) indicates that the stimulation directly affected subcortical structures other than the STN in some patients. Besides stimulation intensities, taking locations of active electrode contacts into account in future studies is therefore vital to understand the role of this excessive stimulation.

### Limitations

A possible limitation of the present approach constitutes its reliance on hemodynamic low-frequency oscillations as systemic physiological oscillations (e.g. Mayer-waves^57^) may partly contribute to the signal variance in the frequency band used here. However, there is increasing evidence that this signal component reflects neuronal activity^58^ and conveys information about functional connectivity^59,60^. In addition, we recently demonstrated the robustness of the present approach against physiological noise^35^. The network connections reconstructed here are hence unlikely to reflect mere physiological artifacts but instead represent (direct or indirect) signaling pathways between neuronal populations.

A pivotal question that remains is whether the disruption of the prefrontal hierarchy by STN-DBS is indeed causal for cognitive decline. The present findings suggest that receiving STN-DBS in a more advanced stage of Parkinson’s disease may increase the risk of cognitive side-effects because the stimulation disturbs the hierarchical organization in the PFC. Furthermore, the relevance of this hierarchical organization in the PFC for cognitive functioning is demonstrated in the Supplementary Model S2. Yet, the present data do not allow to conclude that effects of STN-DBS on cognitive performance are mediated by its remote effects on the PFC. Resolving this issue would require to simultaneously conduct cognitive assessments and fNIRS measurements repeatedly ON and OFF STN-DBS, which would however likely be confounded by the patients’ motor symptoms and challenged by psychometric issues^61^.

Although PD patients were assessed on medication adhering to their usual medication to ensure dopamine levels being as stable and as physiological as possible, it cannot be fully excluded that changes in dopamine over time may have at least partly contributed to the differences ON vs. OFF STN-DBS observed in Model 1. However, these STN-DBS effects on the functional network structure in the PFC were replicated in the second (later) ON stimulation measurement (Model 2; cf. Fig. 3 top and bottom row), thus strongly suggesting that the modulation of the rostro-caudal gradient in the PFC was driven by the experimental variation of the STN stimulation and not by changes in dopamine levels over time.

## Conclusion

The present study not only provides novel insights into the remote network effects of STN-DBS but also offers a completely new perspective on the potential neurophysiological mechanisms underlying cognitive and psychiatric side effects of STN-DBS in Parkinson’s disease. Specifically, we demonstrated that the rostro-caudal hierarchy in the PFC is compromised by STN stimulation in patients who underwent electrode implantation after longer disease duration. In contrast, stimulation enhanced the prefrontal hierarchy in patients who received DBS early in the course of their disease. In addition, stimulation intensities and dopaminergic drug dosages predicted the strength of the prefrontal hierarchy. Taken together, by allowing to directly monitor the remote network effects of STN-DBS the novel approach applied here might provide a promising opportunity for future refinements of DBS in the STN and beyond in terms of a methodological foundation for individually tailored optimization and adjustment of stimulation parameters.

## Methods and materials

The study protocol was approved by the ethics committee of the University of Freiburg (vote 410/11) and registered at German Clinical Trials Register (DRKS, www.drks.de, identifier DRKS00003530, date of registration: 10/02/2012). The study was carried out in accordance with the Declaration of Helsinki and the guidelines of the local ethics committee on research involving human participants. All participants gave written informed consent prior to participation.

### Participants and procedures

Twenty-six patients with idiopathic Parkinson’s disease and implanted STN-DBS participated in the present study. To control for hemispheric differences in disease severity, two patients that had no record of the side of disease onset were excluded from the present analyses. Following a neuropsychological assessment (including the Tower of London task, see below), patients were rated by the UPDRS-III and underwent the fNIRS measurements. After the first fNIRS measurement (12 min, ON stimulation), the stimulator was switched off for approximately 2 h. Of the 24 included patients (6 females, mean age ± SD 61.5 ± 9.9 years), 2 dropped out during this period. The second fNIRS measurement was conducted OFF stimulation for 12 min and was preceded by another UPDRS-III assessment. Four further patients dropped out after the measurement OFF stimulation. In order to capture the transition from OFF to ON stimulation, we conducted a third measurement immediately after the stimulator was switched on again. Taken together, three 12-min fNIRS measurements were acquired (Fig. 1a): first ON stimulation (n = 24), thereafter in steady-state OFF stimulation (after 2 h of rest without stimulation; n = 22) and finally in the transition state immediately after the stimulator was switched back on (ON2, n = 18). During all measurements, patients were watching muted movie parts from the nature documentary ‘Earth’^62^; the order of the movie parts was balanced across patients. To ensure dopamine levels to be as stable and as physiological as possible, patients were told to adhere to their usual medication on the day of study participation and were examined during medical ON periods (see Supplemental Table S1 for patient characteristics; cf. Fig. 1a for experimental workflow). As a control group, 24 healthy adults were matched to the patients in terms of age and sex (Supplementary Fig. S1) and underwent a 12-min fNIRS measurement during passive watching of a nature documentary (see Supplementary Model S1 for results and details of healthy controls). Cognitive functioning of patients and healthy controls was assessed with the Tower of London planning task^63^ (see Supplementary Methods for details) which substantially relies on the PFC^64^; planning accuracy was used to predict resting-state directed functional connectivity in Supplementary Model S2 to address the role of prefrontal networks for cognitive abilities.

### Acquisition of fNIRS data

Multi-channel fNIRS was used to record brain activity in the PFC (Fig. 1a) and to explicitly test the hypothesis of rostro-caudally directed interactions as it sampled activation in the PFC at a sufficient temporal resolution of 10 Hz^65^ and at a spatial resolution that ensured separation of rostral, middle, and caudal PFC (Fig. 1e). FNIRS data was acquired using an ETG-4000 optical topography system (Hitachi Medical Systems, Japan). Spatial optode arrangement was derived from the system’s 3 × 11 grid configuration consisting of 17 emitters and 16 detectors. We modified this probe set by placing 12 emitters and 13 detectors on the forehead (interoptode distance of 3 cm resulting in a diagonal channel distance of 2.1 cm), resulting in 38 channels evenly distributed over the PFC (Fig. 1e). Unused emitter optodes were covered by black caps to avoid crosstalk; recordings were performed in a room without windows and the room lights were switched off during measurements. Grid placement over PFC was standardized across patients (I) by aligning the grid center to the sagittal midline and (II) by positioning the lower center optode at a distance of about 1.5 cm above the nasion. Presentation of the nature video and on-/offset of simultaneous fNIRS recordings were controlled by NBS Presentation software (version 12.2; Neurobehavioral Systems Inc., CA). To prevent artifacts during fNIRS measurement due to head movements, participants’ heads were stabilized using a chin rest. Raw data of light intensity changes were converted into hemoglobin concentration changes by in-house Matlab software (version 2012b, The MathWorks, Natick, MA, USA, unpublished tool box) using the modified Beer-Lambert law^66^. Due to the absorption of interfering hairs or saturation of detectors some channels did not contain any signal. The respective time series were interpolated from the surrounding channels using the Matlab 4 griddata method. This affected a total of 15 channels in 9 datasets (out of 2432 channels in 64 datasets; 0.6% of channels). In order to remove motion-induced artifacts, we applied the correlation-based correction method developed by Cui et al.^67^. This method not only effectively removes motion artifacts, but also increases the contrast-to-noise ratio. The resulting data for oxygenated and deoxygenated hemoglobin are perfectly anticorrelated and therefore have identical spectral properties. The time series were standardized for Granger-causality analysis. We refrained from applying further artifact correction methods like filtering or resampling, because such data preprocessing has been shown to produce false positives in Granger-causality analysis^68^. Furthermore, we used a frequency-domain measure of Granger-causality (see below) that allowed avoiding frequency bands prone to physiological noise introduced by respiration and heart-beat (see also Refs.^31,35^). Further information on the validity of the here applied fNIRS approach on directed functional connectivity can be found in Refs.^31,35^.

### Analyses of directed interactions

Directed coherence (DC)^69^ as an implementation of Granger-causality^70^ was used to capture the intrinsic functional organization of the PFC and directed interactions between its rostral and caudal parts. DC was estimated using the frequency domain multivariate analyses toolbox (www.fdm.uni-freiburg.de/Toolboxes/fdma-toolbox). For DC calculation, a vector autoregressive model was fitted with a model order of 20, corresponding to the past 2 s of the time-series and providing a frequency resolution of the DC estimate of 0.5 Hz. Note, however, that for the Fourier transformation the autoregression coefficients were zero-padded to the length of the time series in order to smooth the spectral estimate. As functional connectivity between different brain areas is apparent in low frequency oscillations^59^, we chose the frequency band between 0.06 and 0.12 Hz and used the maximum DC value in this band for further analysis (see also Refs.^31,35^).

### DBS electrode localizations

Preoperative T2-weighted MRI and CT scans as well as postoperative axial slabs of CT scans focusing on the subcortical volume around the electrode tips were acquired. Details on imaging parameters varied across patients and can be requested from the authors. Using BRAINSfit software^71^ as implemented in 3D slicer (version 4.6.0; www.slicer.org), postoperative CTs were linearly co-registered and fused to preoperative CTs. The fused images were then again linearly co-registered to preoperative MRIs. Preoperative MRIs were nonlinearly warped into International Consortium for Brain Mapping (ICBM) 152 2009b nonlinear asymmetric space using the SyN approach as implemented in Advanced Normalization Tools^72^. Using the Lead-DBS software^73^ (version 1.5.1; www.leadsuite.io), electrode positions were reconstructed hybridly in native and standard stereotactic space. Renderings of electrode positions in relation to the STN are provided in Supplementary Fig. S4, showing that implanted electrodes mainly resided within the posterior, i.e. the motor subdivision of the STN.

### Estimation of the volume of activated tissue (VAT)

To construct a conductor model of the DBS electrode and surrounding tissue, the Medtronic DBS electrode 3391 model was discretized into voxels of 0.22 mm isotropic size. The electrode defined the mid-axis of a volume 101 × 101 × 90 voxels in size. The rest of the space was filled with gray and white matter. Gray matter was defined by the structures STN, red nucleus and internal/external pallidum as specified by the DISTAL atlas^3^ which exactly corresponds to the ICBM 2009b nonlinear template used for normalization. The remaining space was declared as white matter. The volume was converted into a hexaedral mesh using FieldTrip software^74^; www.fieldtriptoolbox.org). Conductivities of 0.33 and 0.14 S/m were assigned to gray and white matter respectively. These values are commonly used in neuroscientific modeling studies^75^. For the platinum/iridium contacts and insulated parts of the electrodes, values of 10^8^ S/m and 10^–16^ S/m were used, respectively. A forward model to obtain the voltage distribution was solved using the SimBio toolbox (Ref.^76^; https://www.mrt.uni-jena.de/simbio/index.php/Main_Page). In case of monopolar stimulation, the surface of the cubic model was used as the cathode. This generated a gradient denoting the voltage distribution for each hexaedral element of the model. The gradient was thresholded for magnitudes > 0.2 V/mm^2^ to define the VAT. The VATs in both hemispheres were substantially correlated (Pearson's r = 0.55, p = 0.007) and were therefore averaged and used as a single predictor in the statistical analysis.

### Statistical analyses

DC and between-subject covariates were analyzed in linear mixed effects models using the lme4 package^77^ (version 1.1–14) for R statistics (version 3.4.2; http://cran.r-project.org). According to extant models of prefrontal organization^22,23^, we expected influences predominantly in rostro-caudal direction and therefore selected the connections between 4 rostro-caudally adjacent fNIRS channels in 4 parallel rows within each hemisphere for statistical analyses (i.e., resulting in 12 connections per hemisphere; Fig. 1e). Hemispheres were classified into ‘ipsilateral’ and ‘contralateral’ with respect to the hemisphere of disease onset. Two linear mixed effects models were computed as summarized in Table 1. Models were analyzed following a top-down procedure^42^: Higher-order fixed-effects interaction terms that did not reach significance (i.e. p > 0.05) were identified using the anova method (Type III F-statistics with Satterthwaite's approximation of degrees of freedom) implemented in the lmerTest package^78^ (version 2.0-33) and successively removed to yield a parsimonious model^42^ (see Table 1 for the highest-order terms of the initial models and Supplementary Table S2 for the significant highest-order terms of the parsimonious models). Models were fitted with random intercepts for (I) the interaction between *participant* and *stream* (identifying channel rows in rostro-caudal direction) and (II) the interaction between *participant* and *level* (identifying adjacent channel pairs—i.e. connections—along the rostro-caudal axis) using maximum likelihood estimation. Post-hoc comparisons and calculation of confidence bands were performed using the lsmeans package^79^ (version 2.27-2). Multiplicity adjustment was applied using Tukey’s method.

## Supplementary Information

Supplementary Information.
