# Supplementary material for: The rostro-caudal gradient in the prefrontal cortex and its modulation by subthalamic deep brain stimulation in Parkinson’s disease
Source: Sci Rep. 2021 Jan 22;11:2138. doi: 10.1038/s41598-021-81535-7 (PMC7822958; doi:10.1038/s41598-021-81535-7)
Supplement: Supplementary file 1 — Supplementary Information. [file 41598_2021_81535_MOESM1_ESM.docx]

**The rostro-caudal gradient in the prefrontal cortex and its
modulation by subthalamic deep brain stimulation
in Parkinson’s disease**

*Supplemental Information*

F. Konrad Schumacher, PhD, ^1,2,3,4,10,11^, Lena V. Schumacher, PhD, ^1,3,5,10^, Florian Amtage, MD, ^1,3,10,11^, Andreas Horn, MD, PhD, ^6^, Karl Egger, MD, ^2,3,10^, Tobias Piroth, MD, ^1,7,10^, Cornelius Weiller, MD, ^1,3,10,11^, Björn O. Schelter, PhD, ^8,11^, Volker A. Coenen, MD, ^9,10,11^, Christoph P. Kaller, PhD, ^1,2,3,10,11^

^1^ Dept. of Neurology, Medical Center – University of Freiburg, Germany | ^2^ Dept. of Neuroradiology, Medical Center – University of Freiburg, Germany | ^3^ Freiburg Brain Imaging Center, University of Freiburg, Germany | ^4^ Faculty of Biology, University of Freiburg, Germany | ^5^ Medical Psychology and Medical Sociology, University of Freiburg, Germany | ^6^ Department of Neurology, Movement Disorders and Neuromodulation Unit, Charité – University Medicine Berlin, Germany | ^7^ Kantonsspital Aarau, Switzerland | ^8^ Institute for Complex Systems and Mathematical Biology, University of Aberdeen, UK | ^9^ Dept. of Stereotactic and Functional Neurosurgery, Medical Center – University of Freiburg, Germany | ^10^ Faculty of Medicine, University of Freiburg, Germany | ^11^ BrainLinks-BrainTools Cluster of Excellence, University of Freiburg, Germany

**Table of Contents**

[Supplemental Methods 2](#_Toc57631047)

[The Tower of London (TOL) planning task 2](#_Toc57631048)

[Gray matter volume in the PFC 2](#_Toc57631049)

[Supplemental Analyses 3](#_Toc57631050)

[Model S1: The rostro-caudal gradient is apparent in healthy controls 3](#_Toc57631051)

[Figure S1. Statistics for matching of participants. 4](#_Toc57631052)

[Figure S2. Granger-causality analysis of directed interactions reveals a rostro-caudal hierarchical organization in the PFC of healthy controls 5](#_Toc57631053)

[Model S2: Connectivity within the rostral PFC is associated with planning performance 5](#_Toc57631054)

[Figure S3. Rostrally directed influences exerted by the mid-lateral PFC are associated with better planning abilities 7](#_Toc57631055)

[Model S3: Connectivity is independent of gray matter volume 9](#_Toc57631056)

[Figure S4. Electrode positions. 10](#_Toc57631057)

[Supplemental Tables 11](#_Toc57631058)

[Table S1. Individual clinical data for Parkinson’s patients. 11](#_Toc57631059)

[Table S2. Estimates and contrasts of interest for significant fixed-effects terms 14](#_Toc57631060)

[Table S3. Correlation coefficients with p-values for covariates 16](#_Toc57631061)

[Supplemental References 17](#_Toc57631062)

## Supplemental Methods

### The Tower of London (TOL) planning task

The TOL-Freiburg version^1^ is a normed, computerized version of the original TOL task^2^ and measures participants’ visuospatial planning abilities. It demands that a start state of three balls distributed on three pegs of unequal height (with a maximum capacity for holding three, two, or one balls, respectively) be transformed into a predefined goal state in the minimum number of moves possible while adhering to the following rules: (I) only one ball may be moved at a time, (II) balls cannot be placed outside of the pegs, (III) only the topmost ball on each peg can be moved, (IV) balls cannot be placed on a peg that already holds its maximum number of balls, (V) solutions should be planned in advance of moving any balls. The Freiburg version of the TOL features a structurally balanced problem sets wherein three major determinants of problem difficulty are systematically varied across the problems which have a minimum move length of three, four, five, or six moves (four three-move problems, eight problems for the other move lengths). After written instructions on screen and a set of three two-move practice problems, the experimental problem set is initiated. The three-move problems are used for initial adjustment to the task and are not subjected to analyses. A time limit of 1 minute is imposed for each problem; after three consecutive violations of the time limit, the task is automatically aborted. The TOL’s accuracy score, calculated as the number of problems that were solved within the minimum number of moves divided by the overall number of trials (n=24), has good psychometric properties^3^ and was thus used as the measure of planning performance here. For the present analysis (Supplemental Model S2) accuracy was adjusted for age by using the percentile ranks based on a norm sample of 296 adults (129 male, age range: 16-84 years)^1^.

### Gray matter volume in the PFC

Pre-operative contrast-enhanced MRI scans were used for volumentry as this was the modality most completely available. MRI scans were processed using the Computational Anatomy Toolbox (CAT12)^12^ and PFC gray matter volume for each hemisphere was estimated as the sum of the superior, middle, and inferior frontal gyrus as defined in the LONI Probabilistic Brain Atlas^13^. Images lacking the appropriate quality for processing were excluded. To this end, image quality was assessed by means of the Image Quality Rating (IQR) derived from the brain tissue segmentation using the CAT12 toolbox. The IQR metric is a continuous index that scales between 0 and 100% and is graded from A+ to F which corresponds to an image quality from 100% to 50% (and below), respectively. Gray matter volume was analyzed in Supplemental Models S3.

## Supplemental Analyses

### Model S1: The rostro-caudal gradient is apparent in healthy controls

In Supplemental Model S1, we tested the hypothesized predominance of rostro-caudally directed interactions in the PFC in a sample of matched healthy controls (see Supplemental Figure S1 for matching statistics). The strength of influences (estimated by DC) in rostro-caudal and caudo-rostral direction between adjacent fNIRS channels (illustrated in Fig. 1e in the main text) was entered as the dependent variable in a linear mixed model. The exhaustive model included the predictors *direction of influences*, *hemisphere*, and *age*, as well as all possible interaction terms. Age and hemisphere were included as additional factors in Supplemental Model S1 to ensure comparability to the analyses of Parkinson’s patients (Model 1, main text) in which we sought to assess the side and the timing of disease onset.

The only significant fixed effect in Supplemental Model S1 was exerted by the factor *direction of influences* and – in accordance with the hierarchical model of prefrontal organization – demonstrated a predominance of influences in rostro-caudal direction (F(1,1080) = 39.2, p < .001, see Supplemental Table S2 for an overview of significant fixed effects of all models, estimates and post-hoc tests). Model estimates for the effect of direction are shown in Supplemental Figure S2 (bar plot); the DC values incorporated in the model are visualized topographically on a standard brain surface, i.e. a surface region is colored according to the influence exerted on it by the rostrally (left brain) and caudally (right brain) neighboring region (indicated by arrows).

Topographic visualizations in Supplemental Figures S2 confirmed the overall rostro-caudal pattern of hierarchical processing in the PFC but also revealed deviations. In particular, the region exerting the strongest influences on and receiving the weakest influences from other regions appeared to be mid-lateral PFC, with influences not only toward caudal but also toward rostral PFC. Rostral areas in turn appeared to exert only weak influences on mid-lateral PFC. Thus, even though the major proportion of influences was directed rostro-caudally, data visualization strongly suggested that the main causal source was the mid-lateral PFC. Although at first glance partly contrary to previous conceptualizations^4,5^, this finding is in line with recent work suggesting that the apex of the hierarchy does not reside in the frontal pole but in the mid-dorsolateral PFC^6–8^. Taken together, Supplemental Model S1 established that Granger-causality analyses of directed interactions closely resemble the suggested prefrontal hierarchical organization and thus may act as a proxy for PFC functional integrity.

Figure S1. Statistics for matching of participants.

Healthy controls (HC) were matched to Parkinson’s patients (PD) to simultaneously minimize sex (left panel) and age (right panel) differences between groups.


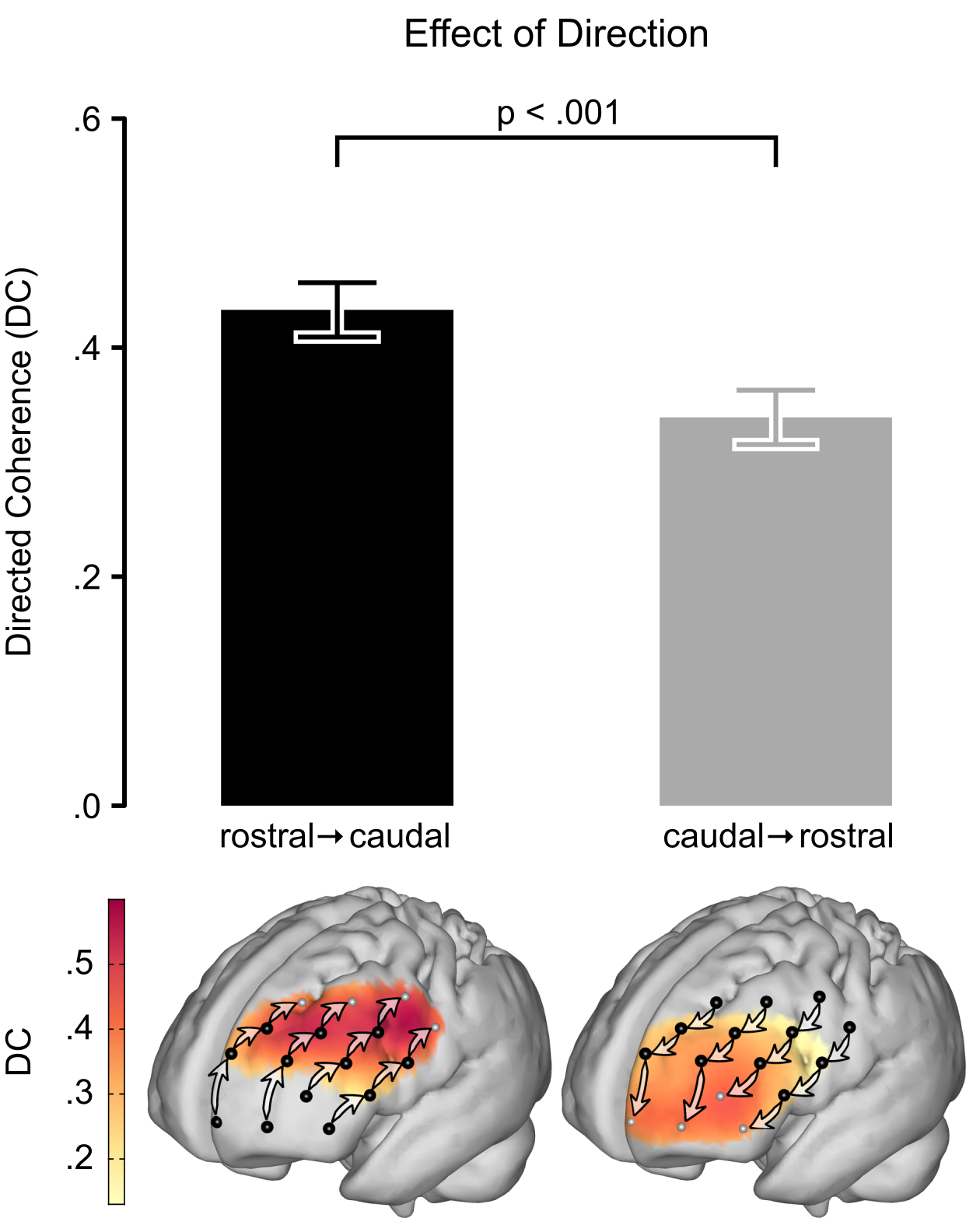


Figure S2. Granger-causality analysis of directed interactions reveals a rostro-caudal hierarchical organization in the PFC of healthy controls

**(Model S1).**

Analyses in the healthy controls showed that influences from rostral to caudal PFC were stronger than from caudal to rostral (top panel). For cortical projections (bottom panel), directed coherence (DC) values were averaged across hemispheres and projected onto the cortical surface to represent the influences from channels (black dots) toward caudally (left brain) and rostrally (right brain) neighboring channels as indicated by arrows. Hot colors signify stronger influences in terms of higher DC values. Projections suggest that the mid-lateral PFC was the main causal source, influencing not only more caudal regions but also the rostral PFC. In return, the most rostral level appeared to exert only little influences over mid-lateral regions. Bars in the top panel represent least square means; error bars indicate 95 % confidence intervals; n = 24.

### Model S2: Connectivity within the rostral PFC is associated with planning performance

As rostro-caudally directed interactions in the PFC and DBS-induced modulations thereof inferred from measurements of intrinsic hemodynamic signals could be purely epiphenomenal, demonstrating a behavioral impact is fundamental to conclude functional relevance. In order to address this issue we again fitted a linear mixed model (Supplemental Model S2) but instead of demographic and treatment-related predictors included the age-corrected percentile rank of accuracy in the Tower of London (TOL) planning task^1^ as a fixed effect. As has been shown by a recent meta-analysis^9^ planning abilities crucially rely on the mid-lateral PFC. We thus tested for differential relations between connectivity and TOL performance across different regions along the rostro-caudal axis by including level as a fixed instead of a random effect. Analyses concerned data from Parkinson’s patients (ON stimulation) and healthy controls. In order to control for the general difference in task performance between groups (Parkinson’s patients: mean percentile rank = 25.1; healthy controls: mean percentile rank = 68.7; t(39.2) = 5.9, p < .001), TOL scores were mean-centered within each group separately and a factor group was included as a fixed effect. The fixed effects structure of the initial model comprised all simple effects and interactions up to the four-way interaction between *group*, *level*, *direction of influences*, and planning *performance* and was again successively reduced by non-significant terms^10^. The parsimonious model contained the three-way interaction between *level*, *direction*, and planning *performance* (F(2,1936) = 4.3, p = .013) and a three-way interaction between *level*, *direction*, and *group* (F(2,1936) = 5.0, p = .007; see also Supplemental Table S2). The latter revealed that, compared to Parkinson’s patients, healthy subjects had a steeper caudally directed gradient on the caudal-most level (p = .049) and a steeper rostrally directed gradient on the rostral-most level (p = .020). The three-way interaction between *level*, *direction*, and planning *performance* further confirmed the regionally specific significance of the rostro-caudal gradient for planning abilities (Supplemental Fig. S3). Contrasts between the directions of influences revealed that higher planning performance was significantly correlated with stronger *rostrally* directed influences on the rostral-most level (p = .003, Supplemental Fig. S3a, top panel). Contrasts between directions on the other levels were not significant. However, a significant simple-effect for planning performance further indicated that stronger directed interactions were generally beneficial for planning abilities (F(1,176) = 11.7, p < .001). This result shows that frontally guided planning abilities benefit from strong influences exerted by the mid-lateral PFC, especially towards rostral PFC. Thus, it further corroborates the notion that the function hierarchy is reversed, i.e. caudo-rostrally directed within the rostral PFC^6–8^.


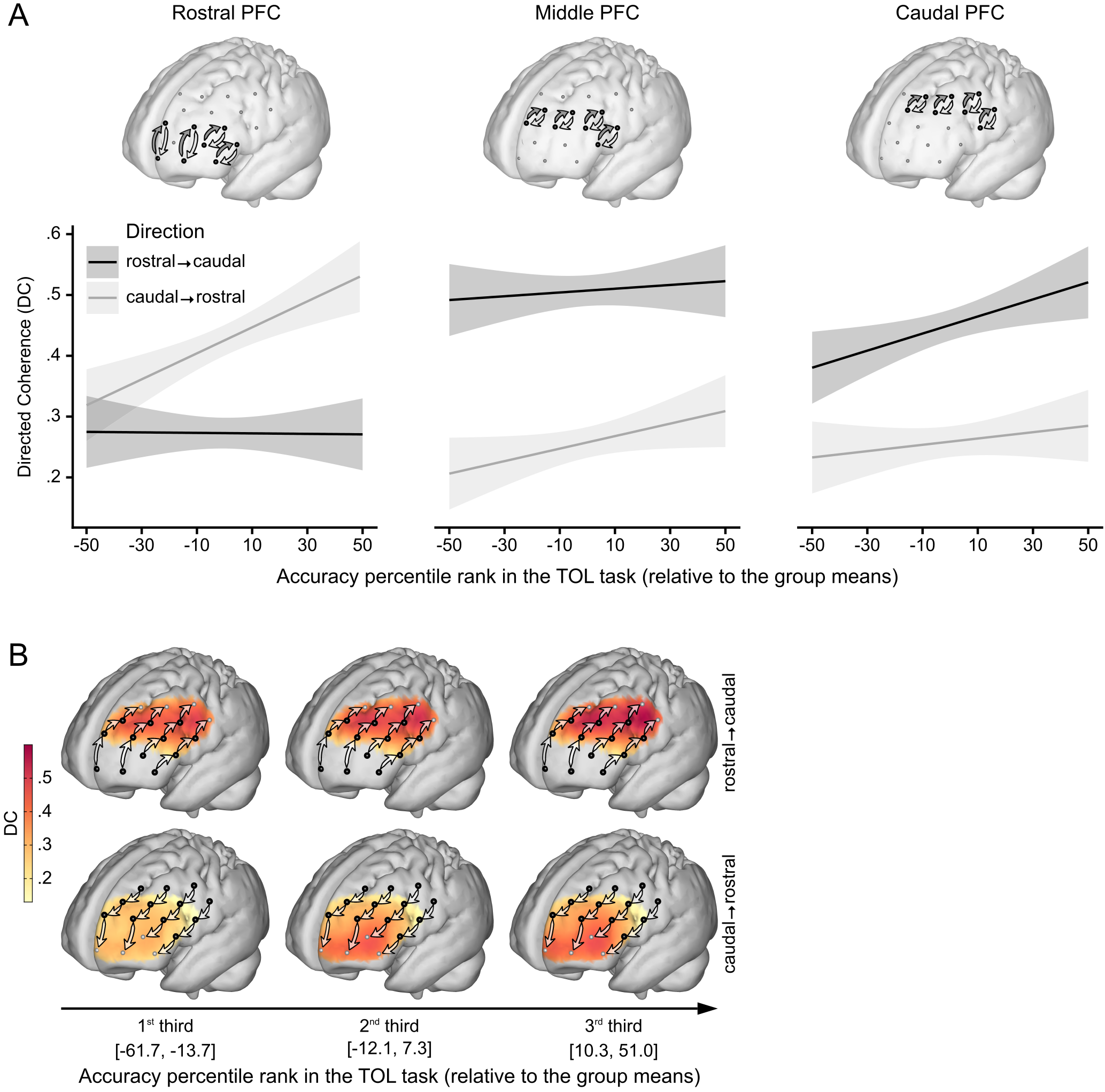


Figure S3. Rostrally directed influences exerted by the mid-lateral PFC are associated with better planning abilities

**in Parkinson’s patients and healthy controls (Model S2).**

The direction of the gradient varies across PFC regions, i.e. within rostral PFC caudally directed influences are predominant, while in middle and caudal regions rostrally directed influences are generally stronger. (a) On the rostral level (left line graph) the difference between the directions of influences was positively correlated with Tower of London (TOL) planning performance such that stronger influences towards the frontal pole were associated with higher TOL planning performance. There was no significant difference between slopes for the two directions on the middle and the caudal level (middle and right line graph). However, the simple effect of TOL planning performance revealed that stronger overall influences, i.e. irrespective of *direction* and *level*, were also associated with better planning performance. Furthermore, the main effect of group showed that healthy controls had stronger overall influences, which is in line with the fact that they also performed better in the TOL task. This confound was controlled for by mean-centering the TOL planning performance separately for both groups. Model predictions are plotted with 95% non-simultaneous confidence bands. (b) Projections of directed coherence (DC) values for influences in rostro-caudal (top row) and caudo-rostral direction (bottom row). Patients and controls were pooled into three groups according to their performance relative to the respective group mean. This resulted in a group of 8 patients and 7 controls with scores below -13, a group of 6 patients and 8 controls with scores between -13 and 10 and a group of 6 patients and 9 controls with scores above 10 relative to the respective group mean. Four patients did not perform the TOL task.

To explore whether a more general measure of cognitive abilities would also be predictive of the rostro-caudal functional network, we refitted Model S2 with the group-wise mean-centered MoCA-score instead of the TOL-score. An outlier with a MoCA-score of 8 was excluded for this analysis. The model using the MoCA-score yielded highly similar results compared to the model using the TOL-score: In particular, a significant three-way interaction between *level*, *direction*, and MoCA (F(2,1936) = 3.2, p = .042) indicated that participants with higher MoCA-scores had stronger rostrally directed connections on the rostral level and stronger caudally directed connections on the caudal level. The corresponding post-hoc test were, however, not significant. Additionally, a significant three-way interaction between *level*, *direction*, and *group* (F(2,1936) = 6.1, p = .002) revealed that on the rostral level the rostrally directed gradient and on the caudal level the caudally directed gradient was significantly steeper in healthy controls compared to PD patients. This is well in line with the first three-way interaction between *level*, *direction*, and MoCA, because healthy controls had higher MoCA scores than PD patients (t(23.2) = 4.1; p < .001). Finally, the simple effect of *MoCA* (F(1,176) = 21.2, p < .001) and the two-way interaction between *group* and *MoCA* (F(1,176) = 4.6, p = .033) again indicated that stronger overall connectivity was beneficial for cognitive functions and that this positive relationship was more pronounced in healthy controls than in PD patients.

Taken together, using the MoCA score to close the link between the functional network in the PFC and cognitive ability corroborated the findings yielded by the analysis using the TOL. As both measures of cognitive functions were only weakly correlated within groups (health controls: r = .12, PD patients: r = .22), this may serve as evidence that the rostro-caudal hierarchical organization of the PFC indeed subserves a broader spectrum of higher-order cognitive functions.

### Model S3: Connectivity is independent of gray matter volume

As disease progression and clinical outcome of STN-DBS in PD patients have previously been associated with cortical atrophy^11^, we analyzed the influence of gray matter volume in the PFC on the directed functional connectivity to preclude that the observed effect of disease duration before DBS implantation was mediated by gray matter structural integrity. For the present analyses, the quality threshold for inclusion of individual images was set to a CAT12 IQR score ≥ 60% which corresponds to an image quality of at least D- (sufficient; see above). Following this procedure, volumetric data were available for 21 patients. Analysis was performed by extending the parsimonious version of Model 1 (main text) by the three-way interaction between direction, stimulation state, and gray matter volume (including all lower-order terms). No fixed effects involving gray matter volume were statistically significant (all p > .076). As the absence of effects in this analysis may be due to relatively low image quality in some patients, analysis was repeated with an image quality threshold of ≥ 70% (satisfactory), reducing the sample size to 14 patients. This model revealed a simple effect of gray matter volume (F(1, 51.8) = 5.3, p = .026) indicating a negative relationship between overall connectivity and cortical structural integrity of the PFC. However, these results should be interpreted with care because the informative value of the analyses were limited by the relatively low quality of some of the available MRI data and the small sample size, respectively.


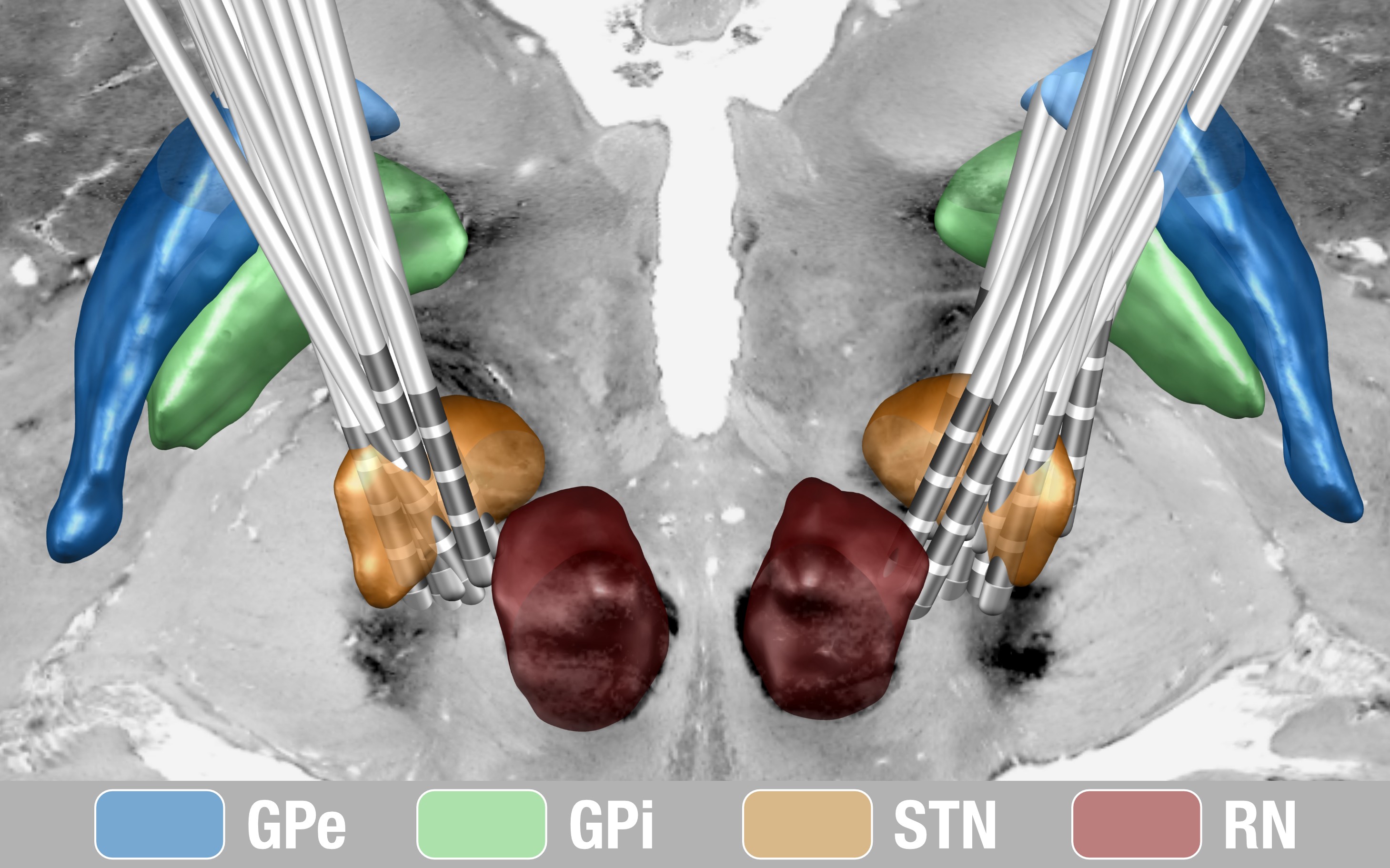


Figure S4. Electrode positions.

. Renderings of electrodes and the STN (orange) in MNI space from posterosuperior view. The electrode contacts reside in the posterior part of the STN, which is associated with motor functions. Abbreviations: GPe, external globus pallidus; GPi, internal globus pallidus; RN, red nucleus; STN, subthalamic nucleus.

## Supplemental Tables

### Table S1. Individual clinical data for Parkinson’s patients.

| **Patient** | **Sex** | **Age (yrs.)** | **Age at onset (yrs.)** | **Disease duration (yrs.)** | | **Body side of onset** | **Disease subtype** | **UPDRS-III** | | **MoCA** |
| --- | --- | --- | --- | --- | --- | --- | --- | --- | --- | --- |
|  |  |  |  | **before implant.** | **since implant.** |  |  | **ON stim** | **OFF stim** |  |
| 1 | M | 71.6 | 50.9 | 9.9 | 10.7 | right | equiv. | 48 | 71 | 29 |
| 2 | F | 69.8 | 52.7 | 16.8 | .3 | right | equiv. | 24 | 39 | 23 |
| 3 | M | 56.3 | 39.5 | 16.2 | .5 | right | equiv. | miss. | miss. | 21 |
| 4 | M | 57.2 | 48.0 | 8.9 | .3 | left | equiv. | 22 | 22 | 26 |
| 5 | M | 66.6 | 56.9 | 7.9 | 1.8 | right | rigid | 17 | N/A | 28 |
| 6 | M | 69.9 | 56.0 | 13.2 | .6 | left | tremor | 14 | 36 | 23 |
| 7 | F | 66.0 | 55.2 | 10.3 | .5 | right | rigid | 24 | 23 | 20 |
| 8 | M | 55.9 | 33.5 | 16.5 | 5.9 | right | rigid | 40 | 51 | 23 |
| 9 | M | 76.6 | 49.9 | 20.0 | 6.7 | left | tremor | 39 | 49 | 17 |
| 10 | M | 65.8 | 51.6 | 13.9 | .3 | left | rigid | 40 | 46 | 25 |
| 11 | M | 49.2 | 37.5 | 3.9 | 7.9 | left | rigid | miss. | miss. | 25 |
| 12 | F | 70.4 | 61.6 | 8.2 | .7 | left | rigid | 27 | 48 | 23 |
| 13 | F | 50.3 | 39.5 | 9.8 | 1.0 | left | rigid | 35 | 37 | 19 |
| 14 | F | 74.4 | 53.6 | 17.3 | 3.4 | left | rigid | 34 | 43 | 8 |
| 15 | M | 69.5 | 62.4 | 6.9 | .2 | right | rigid | 28 | 29 | 20 |
| 16 | M | 55.8 | 47.1 | 7.7 | 1.0 | left | rigid | miss. | miss. | miss. |
| 17 | M | 69.5 | 53.4 | 7.5 | 8.6 | right | tremor | 18 | 48 | 26 |
| 18 | M | 53.5 | 35.3 | 14.4 | 3.8 | right | rigid | 17 | N/A | 27 |
| 19 | F | 61.0 | 43.8 | 10.1 | 7.0 | left | equiv. | 44 | 77 | 27 |
| 20 | M | 66.4 | 50.8 | 15.4 | .1 | right | equiv. | miss. | miss. | miss. |
| 21 | M | 62.9 | 54.3 | 6.6 | 2.1 | right | rigid | miss. | miss. | 24 |
| 22 | M | 55.8 | 45.7 | 9.8 | .4 | right | equiv. | 26 | 41 | 24 |
| 23 | M | 41.4 | 32.9 | 5.3 | 3.2 | right | rigid | 19 | miss. | miss. |
| 24 | M | 40.8 | 29.9 | 10.3 | .6 | right | tremor | 18 | 52 | 30 |
| Mean |  | 61.5 | 47.6 | 11.1 | 2.8 |  |  | 28.1 | 44.5 | 23.2 |
| SD |  | 9.9 | 9.2 | 4.3 | 3.2 |  |  | 10.4 | 14.8 | 4.8 |

Abbreviations: F: female; implant.: implantation; M: male; miss.: missing data; MoCA: Montreal Cognitive Assessment; SD: standard deviation; sym.: symmetric; UPDRS-III: Unified Parkinson's Disease Rating Scale, part III; equiv.: equivalent; yrs: Years.

**Table S1. Individual clinical data for Parkinson’s patients (continued).**

| **Patient** | **LEDD (mg)** | **Stimulator** |  | **VAT (mm³)** | |  |  | **Measurements** | |  |  |
| --- | --- | --- | --- | --- | --- | --- | --- | --- | --- | --- | --- |
|  |  |  | **left STN** | | **right STN** | | **OFF** | | **ON2** | | |
| 1 | 175 | MT Activa PC | 339 | | 175 | | × | | × | | |
| 2 | 590 | MT Activa PC | 126 | | 108 | | × | | × | | |
| 3 | 1180 | MT Activa PC | 174 | | 112 | | × | | × | | |
| 4 | 541 | MT Activa PC | 69 | | 86 | | × | | × | | |
| 5 | 399 | MT Activa PC | 530 | | 179 | |  | |  | | |
| 6 | 375 | BS Vercise | 308 | | 444 | | × | | × | | |
| 7 | 480 | MT Activa PC | 203 | | 173 | | × | | × | | |
| 8 | 680 | MT Kinetra | 107 | | 73 | | × | | × | | |
| 9 | 540 | MT Kinetra | 599 | | 238 | | × | | × | | |
| 10 | 661 | MT Activa PC | 113 | | 113 | | × | |  | | |
| 11 | 762 | MT Activa PC | 71 | | 170 | | × | | × | | |
| 12 | 520 | MT Activa PC | 226 | | 199 | | × | | × | | |
| 13 | 1606 | MT Activa PC | 72 | | 90 | | × | |  | | |
| 14 | 790 | MT Activa PC | 181 | | 162 | | × | | × | | |
| 15 | 1075 | MT Activa PC | 78 | | 99 | | × | | × | | |
| 16 | 570 | MT Activa PC | 63 | | 312 | | × | | × | | |
| 17 | 374 | MT Kinetra | 489 | | 361 | | × | | × | | |
| 18 | 510 | MT Activa PC | 96 | | 123 | |  | |  | | |
| 19 | 525 | MT Activa PC | 140 | | 292 | | × | | × | | |
| 20 | 914 | MT Activa PC | 128 | | 75 | | × | |  | | |
| 21 | 340 | MT Activa PC | miss. | | miss. | | × | |  | | |
| 22 | 530 | MT Activa PC | 97 | | 80 | | × | | × | | |
| 23 | 825 | MT Activa PC | 455 | | 334 | | × | | × | | |
| 24 | 150 | BS Vercise | 357 | | 276 | | × | | × | | |
| Mean | 629 |  | 218 | | 186 | |  | |  | | |
| SD | 324 |  | 165 | | 105 | |  | |  | | |

N.B. VAT for patient #21 could not be estimated because structural MRI data was not available. Abbreviations: BS: Boston Scientific; LEDD: levodopa equivalent daily dose; miss.: missing data; MT: Medtronic; SD: standard deviation; VAT: volume of activated tissue; yrs: Years.

**Table S1. Individual clinical data for Parkinson’s patients (continued).**

| **Patient** | **Stimulation parameters** | | | | | | | | | | | |
| --- | --- | --- | --- | --- | --- | --- | --- | --- | --- | --- | --- | --- |
|  |  | **left STN** | | | |  |  | **right STN** | | | |  |
|  | Intens. | | Pwdth (µs) | Freq. (Hz) | X Y Z  (mm) | | Intens. | | Pwdth (µs) | Freq. (Hz) | X Y Z  (mm) | |
| 1 | 3.8 V | | 60 | 150 | -12.3 -13.7 -6 | | 3.3 V | | 120 | 150 | 14.4 -14.4 -6.4 | |
| 2 | 2.6 V | | 60 | 130 | -14.6 -13 -4.6 | | 2.3 V | | 60 | 130 | 15.1 -11.1 -5.3 | |
| 3 | 2.4 V | | 60 | 130 | -13.5 -12 -5.8 | | 2.2 V | | 60 | 130 | 13.3 -15.6 -7.6 | |
| 4 | 1.8 V | | 60 | 130 | -14.4 -11.9 -4.7 | | 1.8 V | | 60 | 130 | 14.2 -12.9 -5.4 | |
| 5 | 4.4 V | | 60 | 130 | -12.4 -13.7 -7.8 | | 3.5 V | | 60 | 130 | 11.5 -14.8 -7.5 | |
| 6 | 3.7 mA | | 60 | 130 | -13.7 -11.6 -5.3 | | 4.5 mA | | 60 | 130 | 13.9 -11.7 -3.6 | |
| 7 | 3.5 V | | 60 | 130 | -14.1 -11.9 -5.8 | | 2.5 V | | 60 | 130 | 15.9 -11.3 -5.6 | |
| 8 | 2.5 V | | 60 | 130 | -10.8 -14 -9.6 | | 2.0 V | | 60 | 130 | 12.3 -12.5 -7.6 | |
| 9 | 3.9 V | | 60 | 175 | -11.4 -13.9 -9.1 | | 4.0 V | | 90 | 175 | 13.8 -16.5 -7.8 | |
| 10 | 2.5 V | | 60 | 130 | -14 -11.7 -5.4 | | 2.5 V | | 60 | 130 | 12 -12.6 -4.7 | |
| 11 | 2.2 V | | 60 | 160 | -13.6 -13.4 -6.6 | | 2.2 V | | 60 | 160 | 14.7 -12.3 -4.9 | |
| 12 | 3.8 V | | 90 | 130 | -14.9 -11.5 -6.4 | | 3.8 V | | 90 | 130 | 17.4 -11.4 -4.1 | |
| 13 | 2.0 V | | 60 | 130 | -12.7 -10.4 -9.3 | | 2.0 V | | 60 | 130 | 11.8 -11.1 -8.8 | |
| 14 | 3.2 V | | 60 | 130 | -12.3 -12.6 -9.1 | | 3.5 V | | 60 | 130 | 10 -12.2 -12.8 | |
| 15 | 2.5 V | | 60 | 130 | -12.4 -13.1 -4.6 | | 2.5 V | | 60 | 130 | 13.2 -11.4 -6.4 | |
| 16 | 2.0 V | | 60 | 130 | -14.2 -11.8 -6.1 | | 3.0 V | | 60 | 130 | 11.3 -13.9 -8.2 | |
| 17 | 4.1 V | | 60 | 130 | -14.1 -14.7 -6.4 | | 3.4 V | | 60 | 130 | 13.4 -12.7 -6.7 | |
| 18 | 2.8 V | | 60 | 130 | -12.7 -12.6 -5.7 | | 2.7 V | | 60 | 130 | 12.4 -14.1 -6.8 | |
| 19 | 2.9 V | | 60 | 130 | -11.9 -10.6 -7.4 | | 3.0 V | | 60 | 130 | 11.2 -8.6 -6.9 | |
| 20 | 2.1 V | | 60 | 130 | -12.6 -13.9 -6.1 | | 1.8 V | | 60 | 130 | 13.8 -14.3 -5.8 | |
| 21 | 3.3 V | | 60 | 125 | miss. | | 2.0 V | | 60 | 125 | miss. | |
| 22 | 2.3 V | | 60 | 130 | -15.8 -10.9 -3.5 | | 1.8 V | | 60 | 130 | 13.7 -11.9 -6.9 | |
| 23 | 4.0 V | | 90 | 130 | -13.5 -12.2 -6.9 | | 3.7 V | | 90 | 130 | 13 -9.3 -4.7 | |
| 24 | 3.6 mA | | 60 | 149 | -13.2 -13.9 -8.4 | | 3.4 mA | | 60 | 149 | 11.8 -12.7 -8.5 | |
| Mean |  | |  |  | -13.2 -12.6 -6.5 | |  | |  |  | 13.3 -12.5 -6.5 | |
| SD |  | |  |  | 1.1 1.2 1.6 | |  | |  |  | 1.7 1.8 2.0 | |

N.B. X- Y- and Z-coordinates refer to the active contact of the electrode. If multiple contacts were active, respective coordinates were averaged. Electrode positions for patient #21 could not be reconstructed because structural MRI data was not available. Abbreviations: Freq.: frequency; Intens.: Stimulation intensity in volts or milliampere depending on stimulator; miss.: missing data; Pwdth.: pulse width; SD: standard deviation; U: voltage.

### Table S2. Estimates and contrasts of interest for significant fixed-effects terms

**in the linear mixed models.**

| **Model** | **Effect** | **Factor level** |  |  | **Estimate [SE]** | **df** | **p** | **Contrast** | **Estimate [SE]** | **df** | **p** |
| --- | --- | --- | --- | --- | --- | --- | --- | --- | --- | --- | --- |
| 1 PD ON & OFF DBS | dir. *** | rost.→caud. |  |  | .393 [.009] | 155.4 | <.0001 | rost.→caud. - caud.→rost. | .109 [.010] | 1995.4 | <.0001 |
|  |  | caud.→rost. |  |  | .284 [.009] | 155.4 | <.0001 |  |  |  |  |
|  | dir. × LEDD ** | rost.→caud. |  |  | .006 [.011] | 151.2 | .596 | rost.→caud. - caud.→rost. | .037 [.012] | 1995.4 | .002 |
|  |  | caud.→rost. |  |  | -.031 [.011] | 151.2 | .004 |  |  |  |  |
|  | dir. × VAT *** | rost.→caud. |  |  | .026 [.011] | 156.2 | .018 | rost.→caud. - caud.→rost. | .048 [.012] | 1995.4 | .0001 |
|  |  | caud.→rost. |  |  | -.022 [.011] | 156.2 | .043 |  |  |  |  |
|  | dir. × hemisphere * | rost.→caud. | ipsilateral |  | .402 [.012] | 390.9 | <.0001 | contralateral - ipsilateral | -.017 [.015] | 1995.4 | .251 |
|  |  |  | contralateral |  | .385 [.012] | 390.9 | <.0001 |  |  |  |  |
|  |  | caud.→rost. | ipsilateral |  | .271 [.012] | 390.9 | <.0001 | contralateral - ipsilateral | .027 [.015] | 1995.4 | .061 |
|  |  |  | contralateral |  | .298 [.012] | 390.9 | <.0001 |  |  |  |  |
|  | dir. × stim. × disease duration before DBS implantation * | rost.→caud. | ON |  | -.050 [.012] | 368.4 | <.0001 | OFF - ON | .049 [.015] | 2018.3 | .001 |
|  |  |  | OFF |  | -.001 [.012] | 386.5 | .961 |  |  |  |  |
|  |  | caud.→rost. | ON |  | -.002 [.012] | 368.4 | .889 | OFF - ON | -.001 [.015] | 2018.3 | .926 |
|  |  |  | OFF |  | -.003 [.012] | 386.5 | .802 |  |  |  |  |
| 2 PD ON, OFF & 2^nd^ ON DBS | dir. × stim. × disease duration before DBS implantation * | rost.→caud. | ON |  | -.043 [.012] | 462.4 | .0003 | OFF - ON^1^ | .044 [.014] | 2976.7 | .007 |
|  |  |  | OFF |  | .000 [.012] | 487.2 | .987 | ON2 - OFF^1^ | -.039 [.015] | 2982.8 | .029 |
|  |  |  | ON2 |  | -.038 [.013] | 570.0 | .003 | ON2 - ON^1^ | .005 [.015] | 3002.9 | .940 |
|  |  | caud.→rost. | ON |  | .002 [.012] | 462.4 | .836 | OFF - ON^1^ | -.007 [.014] | 2976.7 | .865 |
|  |  |  | OFF |  | -.005 [.012] | 487.2 | .681 | ON2 - OFF^1^ | -.007 [.015] | 2982.8 | .902 |
|  |  |  | ON2 |  | -.012 [.013] | 570.0 | .367 | ON2 - ON^1^ | -.014 [.015] | 3002.9 | .621 |
| S1 HC | dir. *** | rost.→caud. |  |  | .433 [.012] | 189.5 | <.0001 | rost.→caud. - caud.→rost. | .094 [.015] | 1080 | <.0001 |
|  |  | caud.→rost. |  |  | .339 [.012] | 189.5 | <.0001 |  |  |  |  |
| S2 HC & PD ON DBS | level × dir. ×  TOL * | rostral | rost.→caud. |  | -.001 [.013] | 1637.7 | .940 | rost.→caud. - caud.→rost. | -.052 [.018] | 1936 | .003 |
|  |  |  | caud.→rost. |  | .051 [.013] | 1637.7 | .0001 |  |  |  |  |
|  |  | middle | rost.→caud. |  | .007 [.013] | 1637.7 | .568 | rost.→caud. - caud.→rost. | -.017 [.018] | 1936 | .329 |
|  |  |  | caud.→rost. |  | .024 [.013] | 1637.7 | .058 |  |  |  |  |
|  |  | caudal | rost.→caud. |  | .033 [.013] | 1637.7 | .010 | rost.→caud. - caud.→rost. | .021 [.018] | 1936 | .230 |
|  |  |  | caud.→rost. |  | .012 [.013] | 1637.7 | .338 |  |  |  |  |
|  | level × dir. ×  group ** | rostral | rost.→caud. | HC | .272 [.017] | 1637.7 | <.0001 | PD_rost.→caud.-caud.→rost._ - HC_rost.→caud.-caud.→rost._ | .069 [.035] | 1936 | .048 |
|  |  |  |  | PD | .273 [.019] | 1637.7 | <.0001 |  |  |  |  |
|  |  |  | caud.→rost. | HC | .460 [.017] | 1637.7 | <.0001 |  |  |  |  |
|  |  |  |  | PD | .391 [.019] | 1637.7 | <.0001 |  |  |  |  |
|  |  | middle | rost.→caud. | HC | .526 [.017] | 1637.7 | <.0001 | PD_rost.→caud.-caud.→rost._ - HC_rost.→caud.-caud.→rost._ | .027 [.035] | 1936 | .439 |
|  |  |  |  | PD | .489 [.019] | 1637.7 | <.0001 |  |  |  |  |
|  |  |  | caud.→rost. | HC | .290 [.017] | 1637.7 | <.0001 |  |  |  |  |
|  |  |  |  | PD | .226 [.019] | 1637.7 | <.0001 |  |  |  |  |
|  |  | caudal | rost.→caud. | HC | .501 [.017] | 1637.7 | <.0001 | PD_rost.→caud.-caud.→rost._ - HC_rost.→caud.-caud.→rost._ | -.081 [.035] | 1936 | .020 |
|  |  |  |  | PD | .400 [.019] | 1637.7 | <.0001 |  |  |  |  |
|  |  |  | caud.→rost. | HC | .268 [.017] | 1637.7 | <.0001 |  |  |  |  |
|  |  |  |  | PD | .250 [.019] | 1637.7 | <.0001 |  |  |  |  |

N.B. For effects involving continuous predictors (underlined) the estimates represent the slopes for the respective z-standardized variables while for effects only involving discrete predictors the estimates refer to the least square means on the given factor level. *** p < .001; ** p < .01; * p < .05 (for F-statistics of effects see the results section in the main text). ^1^ p-Value adjustment for comparing a family of 3 estimates using Tukey’s method. Abbreviations: caud., caudal; DBS, deep brain stimulation of the subthalamic nucleus; df, degrees of freedom; dir., direction of influences; HC, healthy control group; LEDD, levodopa equivalent daily dose; PD, Parkinson’s patients group; rost., rostral; SE, standard error; stim., stimulation state; TOL, accuracy percentile rank in the Tower of London planning task; VAT, volume of activated tissue.

### Table S3. Correlation coefficients with p-values for covariates

| p-value  Pearson’s r | Age | Age at onset | Disease duration | Disease duration before implant. | Disease duration since implant. | LEDD | VAT | TOL accuracy percentile rank | Gray matter volume |
| --- | --- | --- | --- | --- | --- | --- | --- | --- | --- |
| Age |  | < .0001 | .0586 | .0639 | .5753 | .3783 | .4896 | .0603 | .0042 |
| Age at onset | **.85** |  | .4934 | .8707 | .3699 | .4777 | .6838 | .3846 | .0100 |
| Disease duration | .39 | -.15 |  | < .0001 | .0040 | .6813 | .5420 | .0947 | .3336 |
| Disease duration before implant. | .38 | -.04 | **.79** |  | .7780 | .6427 | .6370 | .3812 | .2208 |
| Disease duration since implant. | .12 | -.19 | **.57** | -.06 |  | .1890 | .1026 | .1491 | .9205 |
| LEDD | -.19 | -.15 | -.09 | .10 | -.28 |  | .0195 | .6388 | .4348 |
| VAT | .15 | .09 | .13 | -.10 | .35 | **-.48** |  | .1924 | .5217 |
| TOL accuracy percentile rank | -.43 | -.21 | -.38 | -.21 | -.33 | -.11 | -.31 |  | .9748 |
| Gray matter volume | **-.60** | **-.55** | -.22 | -.28 | .02 | .18 | .15 | .01 |  |

N.B. Correlations significant on the 5% level are shown in bold. VAT and gray matter volume were aggregated across hemispheres. Abbreviations: implant.: implantation; LEDD: levodopa equivalent daily dose; VAT: volume of activated tissue; TOL: Tower of London task.

## Supplemental References

1. Kaller, C. P., Unterrainer, J. M., Kaiser, S., Weisbrod, M. & Aschenbrenner, S. *Tower of London – Freiburg version*. (Schuhfried, 2012).

2. Shallice, T. Specific impairments of planning. *Philos. Trans. R. Soc. Lond. B. Biol. Sci.* **298**, 199–209 (1982).

3. Köstering, L. *et al.* Assessment of planning performance in clinical samples: Reliability and validity of the Tower of London task (TOL-F). *Neuropsychologia* **75**, 646–55 (2015).

4. Koechlin, E., Ody, C. & Kouneiher, F. The architecture of cognitive control in the human prefrontal cortex. *Science* **302**, 1181–5 (2003).

5. Badre, D. & D’Esposito, M. Is the rostro-caudal axis of the frontal lobe hierarchical? *Nat. Rev. Neurosci.* **10**, 659–69 (2009).

6. Nee, D. E. & D’Esposito, M. The hierarchical organization of the lateral prefrontal cortex. *Elife* **5**, e12112 (2016).

7. Margulies, D. S. *et al.* Situating the default-mode network along a principal gradient of macroscale cortical organization. *Proc. Natl. Acad. Sci. U. S. A.* **113**, 12574–12579 (2016).

8. Badre, D. & Nee, D. E. Frontal Cortex and the Hierarchical Control of Behavior. *Trends Cogn. Sci.* **22**, 170–188 (2018).

9. Nitschke, K., Köstering, L., Finkel, L., Weiller, C. & Kaller, C. P. A Meta-analysis on the neural basis of planning: Activation likelihood estimation of functional brain imaging results in the Tower of London task. *Hum. Brain Mapp.* **38**, 396–413 (2017).

10. West, B. T., Welch, K. B. & Galecki, A. T. *Linear Mixed Models: A Practical Guide Using Statistical Software*. (Chapman & Hall/CRC, 2014).

11. Muthuraman, M. *et al.* Effects of DBS in parkinsonian patients depend on the structural integrity of frontal cortex. *Sci. Rep.* **7**, 43571 (2017).

12. Gaser, C. & Dahnke, R. CAT-A Computational Anatomy Toolbox for SPM. (2016). Available at: http://www.neuro.uni-jena.de/cat/. (Accessed: 25th November 2020)

13. Shattuck, D. W. *et al.* Construction of a 3D probabilistic atlas of human cortical structures. *Neuroimage* **39**, 1064–1080 (2008).
